# Metabolic engineering of *Escherichia coli* for high-level production of violaxanthin

**DOI:** 10.1186/s12934-023-02098-y

**Published:** 2023-06-21

**Authors:** Dong Xinrui, Liu Bo, Bao Yihong, Liu Weifeng, Tao Yong

**Affiliations:** 1 College of Forestry, Northeast Forestry University, No. 26 Hexing Road, Harbin, 150040 Heilongjiang Province People’s Republic of China; 2 CAS Key Laboratory of Microbial Physiological and Metabolic Engineering, State Key Laboratory of Microbial Resources, Institute of Microbiology, Chinese Academy of Sciences, No. 1 Beichen West Road, Chaoyang District, Beijing, 100101 People’s Republic of China; 3 University of Chinese Academy of Sciences, No. 19A Yuquan Road, Shijingshan District, Beijing, 100049 People’s Republic of China; 4 Heilongjiang Key Laboratory of Forest Food Resources Utilization, No. 26 Hexing Road, Harbin, 150040 Heilongjiang Province People’s Republic of China; 5 Microcyto Co. Ltd, Beijing, People’s Republic of China

**Keywords:** Violaxanthin, *Escherichia coli*, Zeaxanthin epoxidase, Metabolic engineering

## Abstract

**Background:**

Xanthophylls are a large class of carotenoids that are found in a variety of organisms and play particularly important roles in the light-harvesting and photoprotection processes of plants and algae. Violaxanthin is an important plant-derived xanthophyll with wide potential applications in medicines, foods, and cosmetics because of its antioxidant activity and bright yellow color. To date, however, violaxanthins have not been produced using metabolically engineered microbes on a commercial scale. Metabolic engineering for microbial production of violaxanthin is hindered by inefficient synthesis pathway in the heterologous host. We systematically optimized the carotenoid chassis and improved the functional expression of key enzymes of violaxanthin biosynthesis in *Escherichia coli*.

**Results:**

Co-overexpression of *crtY* (encoding lycopene β-cyclase), *crtZ* (encoding β-carotene 3-hydroxylase), *and ZEP* (encoding zeaxanthin epoxidase) had a notable impact on their functions, resulting in the accumulation of intermediate products, specifically lycopene and β-carotene. A chassis strain that did not accumulate the intermediate was optimized by several approaches. A promoter library was used to optimize the expression of *crtY* and *crtZ*. The resulting strain DZ12 produced zeaxanthin without intermediates. The expression of *ZEP* was further systematically optimized by using DZ12 as the chassis host. By using a low copy number plasmid and a modified dithiol/disulfide system, and by co-expressing a full electron transport chain, we generated a strain producing violaxanthin at about 25.28 ± 3.94 mg/g dry cell weight with decreased byproduct accumulation.

**Conclusion:**

We developed an efficient metabolically engineered *Escherichia coli* strain capable of producing a large amount of violaxanthin. This is the first report of a metabolically engineered microbial platform that could be used for the commercial production of violaxanthin.

**Supplementary Information:**

The online version contains supplementary material available at 10.1186/s12934-023-02098-y.

## Background

Microbial cell factories have been widely used to produce diverse natural products, because of their advantages of a short production period, environmentally friendly processes, and their ability to utilize sustainable resources [1, 2]. Carotenoids are one of the most important classes of natural pigments with important physiological functions and diverse potential applications [3, 4]. The production of carotenoids using metabolically engineered microorganisms has attracted much attention as an alternative route to the current processes of extraction from natural sources and chemical synthesis. High microbial production of several important carotenoids, including lycopene, β-carotene, and astaxanthin, have been successfully achieved by metabolic engineering approaches [1, 5–7].

Xanthophylls are a class of oxygen-containing carotenoids and widely distributed in plants as the major pigments. Plant-derived xanthophylls, including violaxanthin, antheraxanthin, lutein and zeaxanthin, play important roles in light harvesting and photoprotection in plant tissues [8, 9]. Therefore, plant-derived xanthophylls have great potential applications as natural antioxidants and coloring agents. However, most plant xanthophylls have not been produced successfully using engineered microbial systems. Previous work has revealed that the functional expression of plant-derived enzymes within xanthophylls pathway is the major bottleneck for xanthophylls microbial production [10]. Violaxanthin is an important xanthophyll in high plants and microalgae. Violaxanthin is the key precursor of abscisic acid, fucoxanthin, capsanthin, capsorubin, and β-damascenone [11]. It participates in the plant violaxanthin cycle and is widely distributed in the leaves, flowers, and fruits of ferns, mosses, and seed plants [12]. Compared with β-carotene and lutein, violaxanthin has a stronger antioxidant capacity [13]. Violaxanthin has also been shown to have anticancer, anti-inflammatory, and other biological activities; thus it has great application value in medicines, foods, cosmetics, and other products [14].

Production of violaxanthin using metabolic engineered microorganisms is hindered by the low production level. Violaxanthin can be produced at about 7.3 mg/g DCW in *Saccharomyces cerevisiae* [15]. In *Escherichia coli*, the production level of violaxanthin is less than 1 mg/g DCW [11]. These production metrics are much lower than that of engineered lycopene- or β-carotene-producing strains. Microbial production of violaxanthin with high production performance is still not accessible. Previously, we have constructed a series of *Escherichia coli* strains for the production of carotenoids, including lycopene and β-carotene [16]. We sought to develop an efficient microbial route for the production of violaxanthin using our chassis strains.

**Fig. 1:**
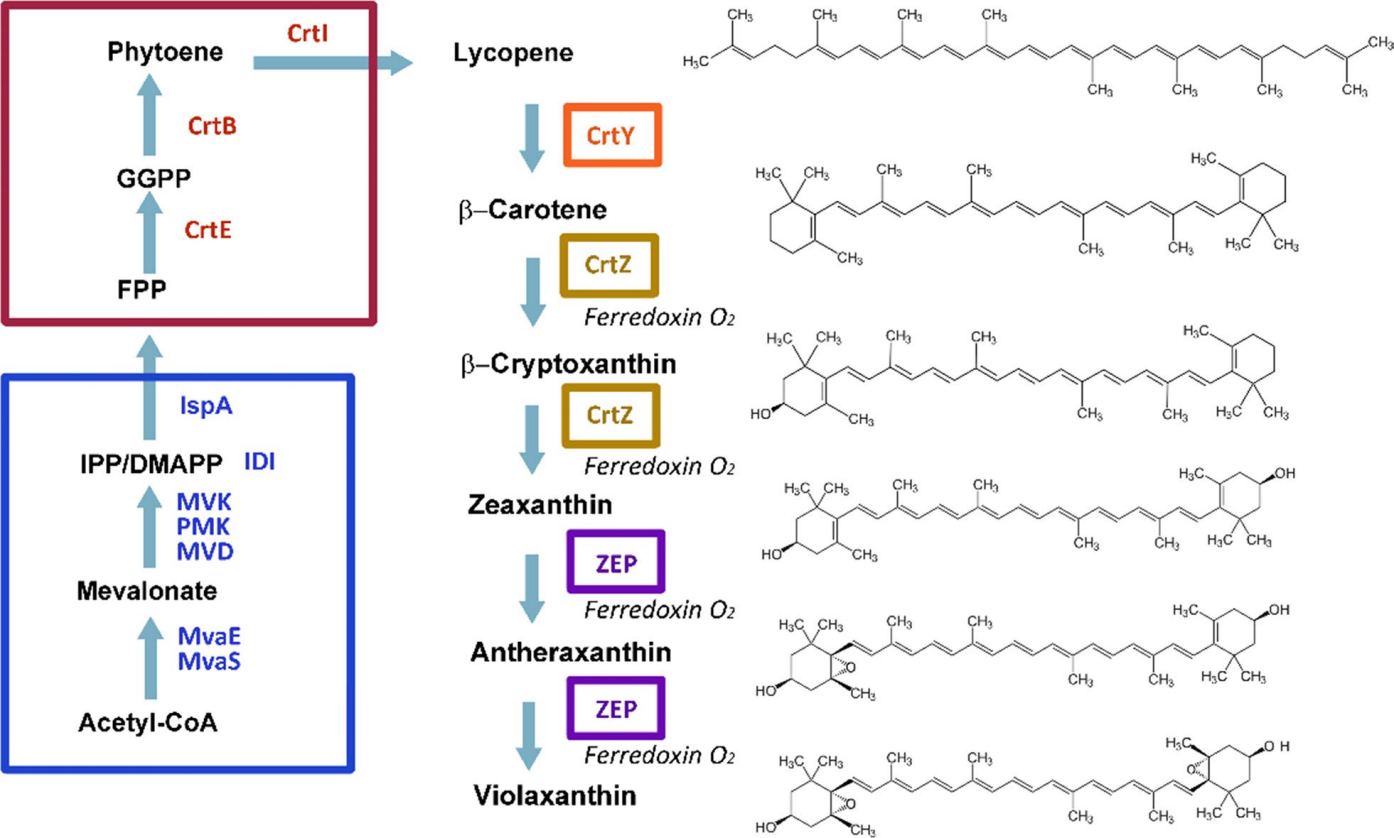
Violaxanthin synthesis pathway in this study. MVA pathway was used to supply the universal precursors IPP and DMAPP (blue box); lycopene-biosynthesis module (red box), lycopene β-cyclase (CrtY), β-carotene 3-hydroxylase (CrtZ), and zeaxanthin epoxidase (ZEP) catalyze the conversion from IPP and DMAPP to violaxanthin. The abbreviation of enzyme names was given

The biosynthesis of violaxanthin requires the universal carotenoid precursors isopentenyl diphosphate (IPP) and dimethylallyl diphosphate (DMAPP), which are supplied by the mevalonate (MVA) pathway or the methylerythritol 4-phosphate (MEP) pathway. The condensation of IPP and DMAPP results in the formation of the intermediates farnesyl diphosphate (FPP, C15) and geranylgeranyl diphosphate (GGPP, C20). Lycopene, the precursor of violaxanthin, is biosynthesized *via* sequential reactions catalyzed by farnesyl diphosphate synthase (FPPS), geranylgeranyl diphosphate synthase (GGPPS,CrtE), phytoene synthase (CrtB), and phytoene desaturase (CrtI). Three further steps are required to synthesize violaxanthin from lycopene. Lycopene is first cyclized to form β-carotene by lycopene β-cyclase (CrtY), and then β-carotene is converted to zeaxanthin by β-carotene 3-hydroxylase (CrtZ). Violaxanthin is finally oxidized to form zeaxanthin by zeaxanthin epoxidase (ZEP) (Fig. 1).

In plants, the activity of hydroxylation and epoxidation steps depend on many factors and conditions, such as electric transfer system and membrane-association. We speculated the activity of these enzymes is limited due to the unfavorable protein expression and condition in heterologous hosts. In this study, we alleviated the bottleneck at the key enzymatic step of violaxanthin biosynthesis pathway in *Escherichia coli*, and developed an optimized strain that can produce large amounts of violaxanthin with minimal byproducts.

## Results

### Development of a chassis strain for violaxanthin biosynthesis

**Fig. 2:**
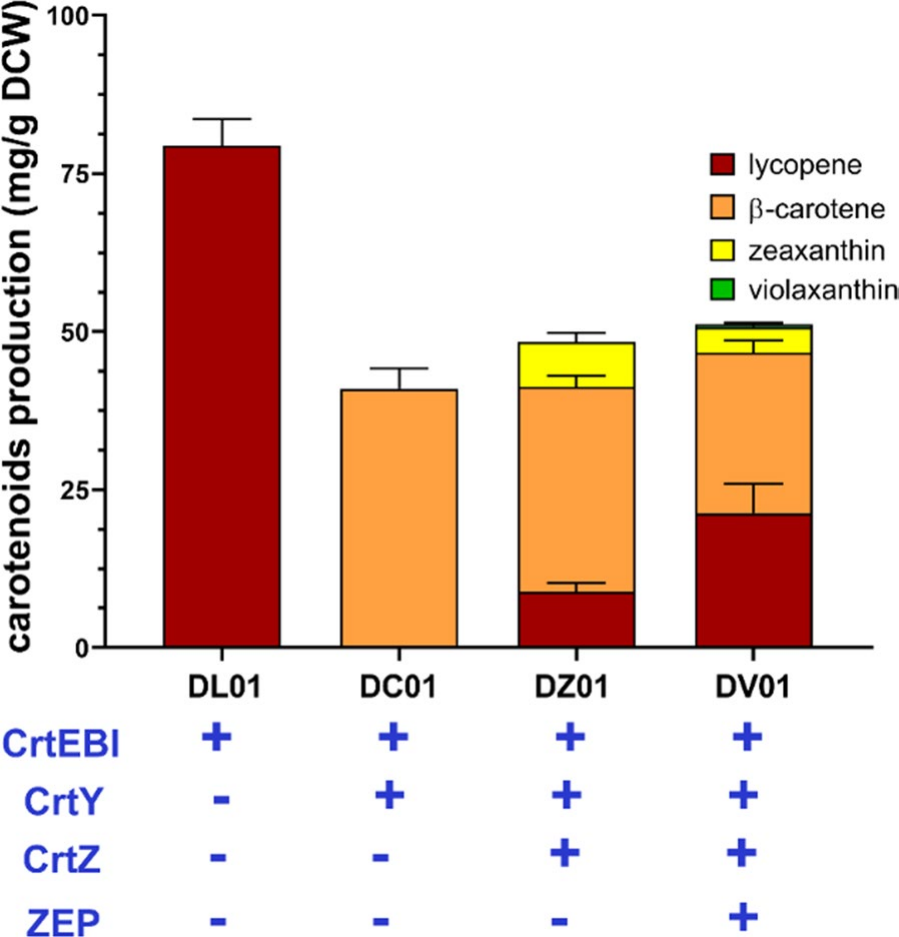
Carotenoids production of lycopene-, β-carotene-, zeaxanthin-, and violaxanthin-producing strain (strain DL01, DC01, DZ01, and DV01, respectively). All strains were cultured in flasks and carotenoids production and content were determined by HPLC after bioconversion for 16 h

First, we sought to optimize a chassis strain that could efficiently supply carotene precursors for violaxanthin synthesis. To simplify the plasmid system, the MVA pathway gene (*mvaS-mvaE-mvk-pmk-mvd-idi*) for IPP/DMAPP synthesis was amplified from the previously constructed plasmids pLE2SK and pSKPMIc and integrated into the genome of *E. coli* strain BW25113 [16]. The *crtEBI* gene was further amplified from plasmid pLY122 to generate lycopene producing strain DL01 [17]. Because the cell growth of all strains in this study was comparable in the flask culture (Additional file 1: Table S6), the strains were mainly evaluated using the carotenoids production of dry cell weight (DCW). Strain DL01 could produce lycopene at 79.43 ± 4.20 mg/g DCW. The *Pantoea agglomerans crtY* gene encoding lycopene cyclase was further introduced into DL01 in a p15A-origin derived plasmid (pYCA01) for the synthesis of β-carotene. The obtained DC01 strain produced β-carotene at 41.00 ± 3.16 mg/g DCW, with no accumulation of the lycopene intermediate. Next, the *Pantoea agglomerans* c*rtZ* gene encoding β-carotene 3-hydroxylase and the *Capsicum annuum ZEP* gene encoding zeaxanthin epoxidase were inserted into a pMB1-origin derived plasmid (pMZP01) and introduced to DC01 (yielding strain DV01). However, DV01 produced very little violaxanthin (0.42 ± 0.37 mg/g DCW), but accumulated large amounts of zeaxanthin, lycopene, and β-carotene (4.10 ± 0.65, 21.32 ± 0.45, and 25.32 ± 1.99 mg/g DCW, respectively). This result implied that simultaneous expression of *crtY*, *crtZ*, and *ZEP* significantly affected their function. Thus, we constructed a *crtZ*-only plasmid (pMZ01) to investigate the functional expression of CrtZ in DC01. The strain harboring pMZ01 produced zeaxanthin at 7.08 ± 1.50 mg/g DCW, but still accumulated lycopene and β-carotene to 8.90 ± 1.34 and 32.36 ± 1.73 mg/g DCW, respectively (DZ01, Fig. 2). Therefore, CrtZ could only convert a portion of β-carotene into zeaxanthin. The activity of CrtY appears to have been impacted by co-expression with *crtZ*. As demonstrated by DC01, lycopene accumulation did not occur without *crtZ* overexpression. However, when *crtY* was co-expressed with *crtZ* in DZ01, it was unable to completely convert all lycopene into β-carotene.

### Optimization of *crtZ* and *ZEP* expression by modifying the plasmid copy number promotes the synthesis of violaxanthin synthesis but leads to accumulation of intermediates

**Fig. 3:**
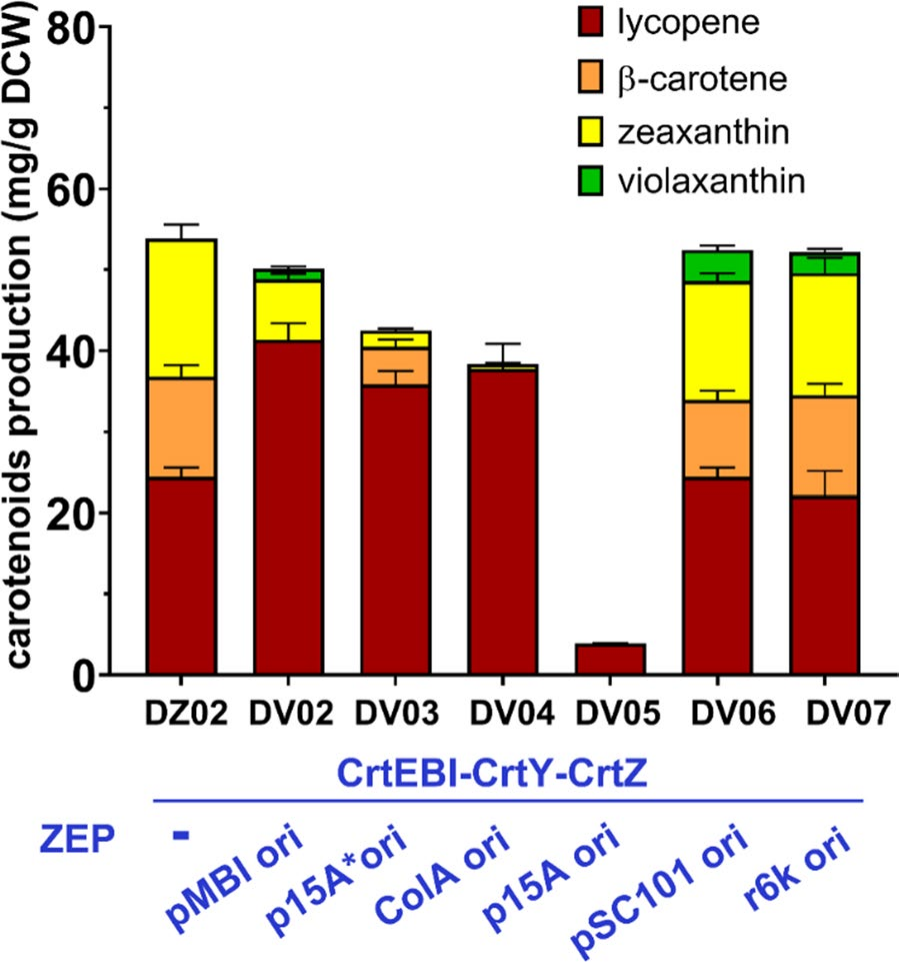
Optimization of the expression of *ZEP* in chassis strain DZ02. *CZEP* was expressed using different plasmids and carotenoid production was investigated. All strains were cultured in flasks and carotenoids production and content were determined by HPLC after bioconversion for 16 h

We suspected that a major bottleneck during strain optimization was the insufficient conversion of β-carotene to zeaxanthin, resulting in low zeaxanthin levels, which limited violaxanthin production. Thus, it was important to increase the zeaxanthin level in the chassis strain. The expression of *crtY* and *crtZ* was then optimized using different expression strategies, including chromosomal expressing or using different plasmids with a variety of replication origins. As shown in Additional file 1: Table S5 and Fig. 3, maximum zeaxanthin production (17.08 ± 1.71 mg/g DCW in strain DZ02) was achieved when *crtY* and *crtZ* were expressed in the chromosome under the control of the P_119_ promoter [18]. The zeaxanthin production in DZ02 was about 141% higher than that in DZ01, and its β-carotene production was decreased to 12.39 ± 1.40 mg/g DCW. To investigate whether the increased zeaxanthin level could improve violaxanthin synthesis, *ZEP* was introduced into DZ02 (strain DV02). Violaxanthin was produced at 1.32 ± 0.27 mg/g DCW in DV02, about 3-fold higher than that in DV01. We further optimized *ZEP* expression by using different plasmids, and found that violaxanthin production was improved to 3.90 ± 0.55 mg/g DCW when *ZEP* was supplied in the pSC101 plasmid (DV06). This result confirmed that an improved zeaxanthin supply can increase violaxanthin synthesis. Meanwhile, a large amount of lycopene accumulated in this strain, indicating that the function of CrtY was still suppressed. The unfavorable expression of *ZEP* appeared to severely exacerbate this effect (DV02-05, as shown in Fig. 3). However, in the two high-violaxanthin-producing strains, DV06 and DV07, the contents of the carotenoid precursors (lycopene, β-carotene, and zeaxanthin) were similar to the parent strain DZ02. To simplify the optimization work, we prioritized optimizing the balance of *crtY* and *crtZ* expression in the chassis strain.

### Selection of engineered *Escherichia coli* with zeaxanthin as a single product after transformation with a promoter library

**Fig. 4:**
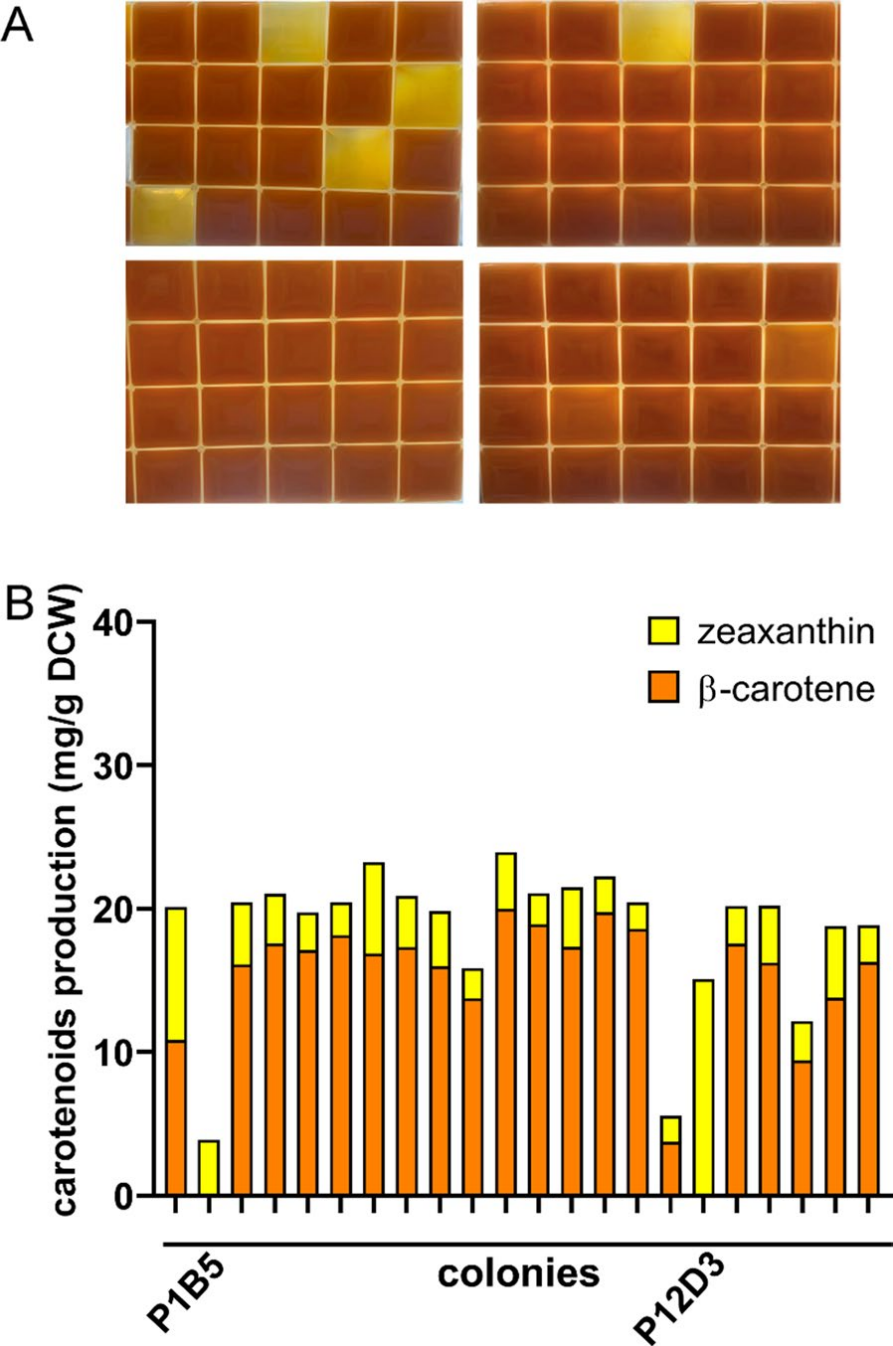
Screening of *E. coli* strains with promoter library driving the expression of *crtY and crtZ*, based on carotenoid production. **A** Screened strains cultured in 24 deep-well plates. Few strains retained their light color in the well-plate culture. **B** Zeaxanthin and β-carotene production of 22 selected strains. Strains were cultured in 24-deep-well plates and carotenoids production and content were determined by HPLC after bioconversion for 20 h

**Fig. 5:**
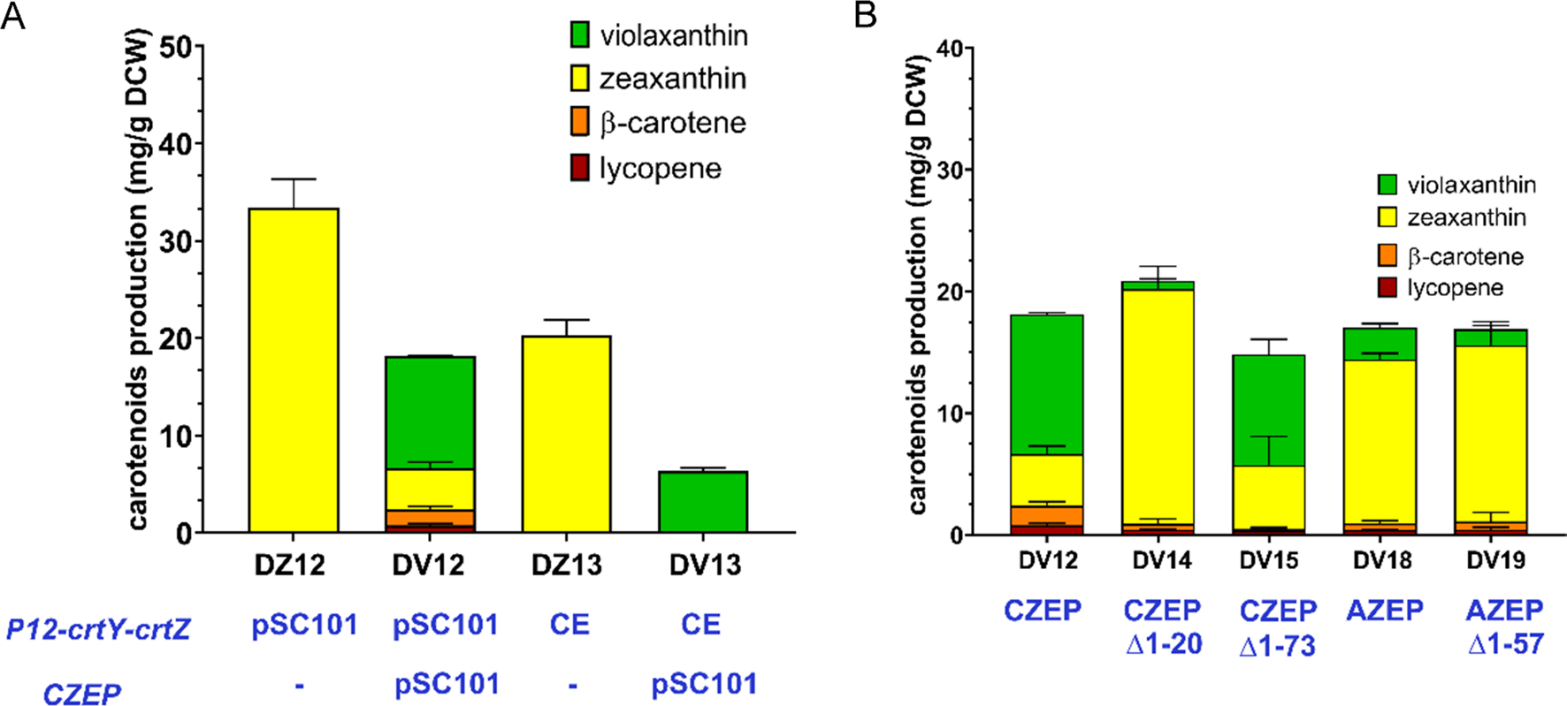
Optimization of violaxanthin biosynthesis by optimizing *ZEP* expression in chassis strains DZ12 and DZ13. **A** Violaxanthin production in DZ12 and DZ13 strains. pSC101below the figure indicates expression in a pSC101 origin plasmid; CE below the figure indicates chromosomal expression. **B** Violaxanthin production in strains harboring *ZEP* genes from different species and their truncated variants. All strains were cultured in flasks and carotenoids production and content were determined by HPLC after bioconversion for 16 h

Next, we sought to fine-tune the expression of *crtY* and *crtZ* by controlling the strength of the promoter to balance the metabolic flux. A promoter library was created by randomizing the key sequences within the core sequence or between − 10 and − 35 regions of two constitutive promoters, P_119_ and P_tac_, using degenerate primers. In addition to the P_119_ promoter, we also used a constitutively strong P_tac_ promoter that had been modified to remove lacI of the original tac promoter (see Additional file 1: Table S4) [18]. The gene fragments of crtY and crtZ were assembled with a mixed library of P_119_ and P_tac_ promoter mutants in pSC101-derived plasmids, allowing for independent control of crtY and crtZ expression under either P_119_ or P_tac_-derived promoters. The plasmid library was then transformed into the lycopene-producing strain DL01 and spread on plate. The potential colonies with favorable expression of *crtY* and *crtZ* were picked based on colony color. The high-zeaxanthin-producing strains might be light yellow color. Up to about 300 colonies among about 5 × 10^3^ colonies on the plates were picked. The selected colonies were then transferred to 24 deep-well plates for the next screening round (Fig. 4A). However, most clones exhibited a deep red color when cultured in deep-well plates, suggesting high lycopene production. Among them, 22 strains that maintained a light yellow color in liquid culture were selected and subjected to further analysis. Almost no lycopene accumulated in the selected strains. However, β-carotene was still the main carotenoid component in most of these strains (Fig. 4B). Few strains with light color in the deep-well plate culture were due to the lower cell concentration. However, two strains could produce zeaxanthin as the single product (The promoter sequences of *crtY* and *crtZ* in these two strains were listed in Additional file 1: Table S4). Among them, strain P12D3 (re-named as DZ12) produced zeaxanthin as the single product with a production of about 15 mg/g DCW in deep-well plate culture. Zeaxanthin production in DZ12 was further confirmed in flask culture, with its production reaching 33.37 ± 2.98 mg/g DCW without accumulation of carotenoid byproducts (Fig. 5A). Therefore, the *crtY-crtZ* expression cluster in DZ12 (*P12-crtY-crtZ* cluster), of which both crtY and crtZ were controlled under P119 promoter mutants, resulted in high-level and specific zeaxanthin production. Consequently, strain DZ12 served as the chassis strain for further optimization. We also attempted to construct libraries based on another strong constitutive P_CPA1_ and RBS to optimize zeaxanthin production, but it was unsuccessful (data not shown).

### Effects of* ZEP *genes and their truncated variants from several photosynthetic eukaryotes on violaxanthin yield

**Fig. 6:**
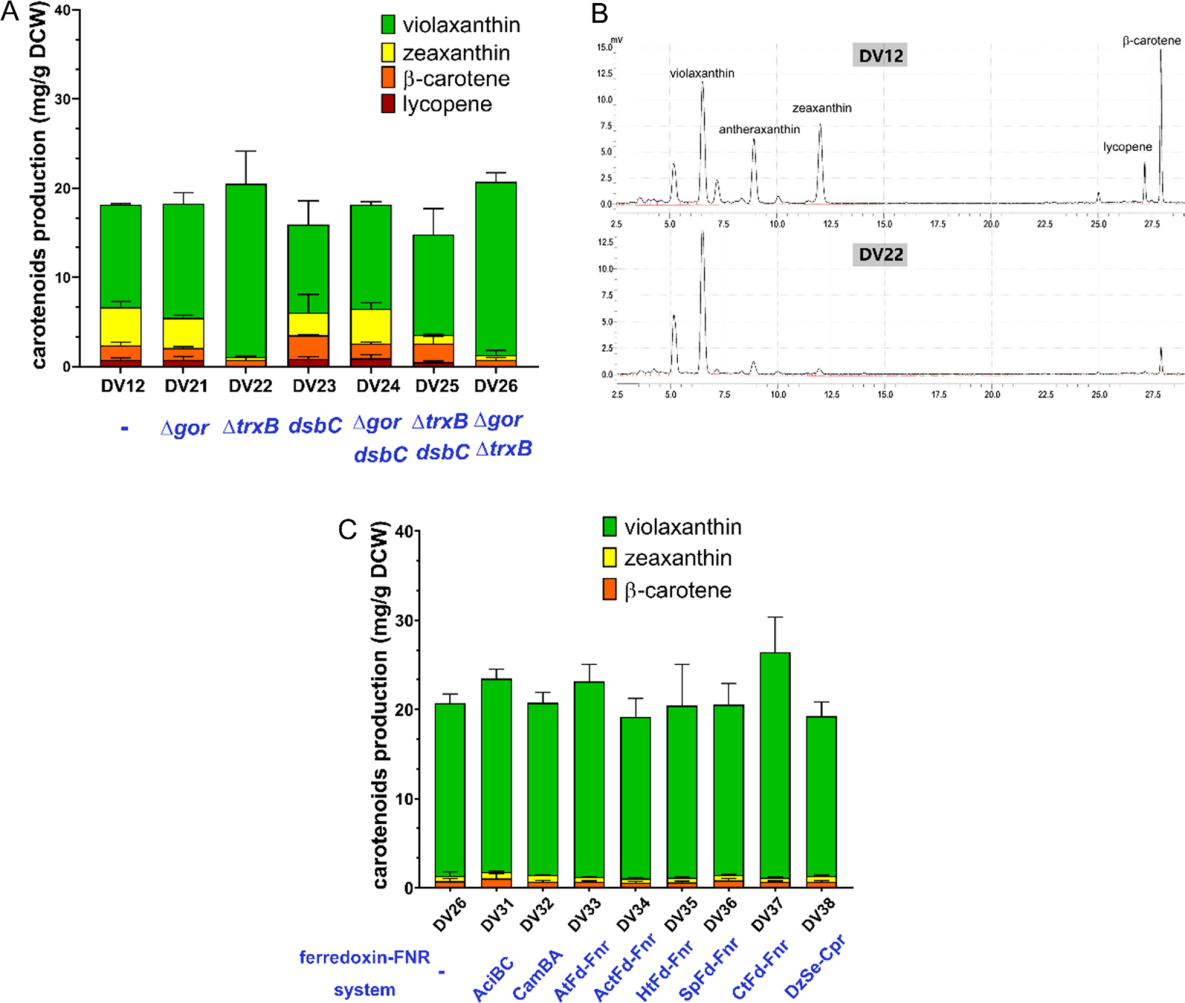
Improving violaxanthin biosynthesis by engineering cytoplasm redox state. **A** Carotenoids production and content of strains after modifying the redox state-related targets. All strains were cultured in flasks and carotenoids production and content were determined by HPLC after bioconversion for 16 h. **B** HPLC analysis of strain DV12 and DV22. **C** Improving violaxanthin biosynthesis by engineering electric transfer systems. All strains were cultured in flasks and carotenoids production and content were determined by HPLC after bioconversion for 16 h

To investigate if strain DZ12 was suitable for violaxanthin synthesis, *ZEP* was inserted into the *P12-crtY-crtZ* cluster-harboring plasmid pSYZ17 of DZ12. The resulting strain DV12 produced violaxanthin at 11.49 ± 0.12 mg/g DCW (Fig. 5). However, DV12 still accumulated zeaxanthin at 4.25 ± 0.66 mg/g DCW, as well as small amounts of lycopene and β-carotene. Antheraxanthin was also emerged accompanying with the high production of violaxanthin in DV12 as detected by HPLC analysis (Fig. 6B. confirmed using HPLC-MS, could not quantified because standard was not available. Production calculated using violaxanthin standard curve was summarized in Additional file 1: Table S6). We also integrated the *DZ12-crtY-crtZ* cluster into the chromosome of DL01 to optimize the chassis strain. The resulting DZ13 strain produced zeaxanthin as the single carotenoid product at 20.24 ± 1.65 mg/g DCW. By using DZ13 as chassis strain, violaxanthin could be produced at 6.32 ± 0.37 mg/g DCW, but no carotenoid byproducts accumulated (Fig. 5). However, to obtain a high production level of violaxanthin, we preferred DZ12 as the chassis host. Thus, DZ12 was used to evaluate the performance of different *ZEP* genes.

Several *ZEP* genes from different plant species are known to be functional in microbial hosts. Thus, we compared the performance of *ZEP* genes from three photosynthetic eukaryotes: *Capsicum annuum* (*CZep*), *Haematococcus lacustris* (*HZep*), *Arabidopsis thaliana* (*AZep*). Furthermore, it was reported N-terminal truncation of ZEP significantly increased its activity [10, 15]. Thus, the effect of N-terminal truncation at different positions was also evaluated. The highest violaxanthin production was still observed in DV12 (expressing the full-length *CZep*). Compared with strain DV12, strain DV18 (expressing the full-length *AZep*) produced much less violaxanthin. However, the truncation of *CZep* lead to remarkable decreases in violaxanthin production, even though the truncated regions corresponded to the plastid transit peptides. This result was in line with previous report [10]. The truncated *AZep* also produced less violaxanthin compared with full length *Azep*. However, this was different with above literature [10]. This probably was due to the translation or protein folding efficiency of AZEP was different in different chassis host and when co-expressed with different heterogenous enzymes. Furthermore, both the full-length *HZep* and its truncated variants were inactive in *Escherichia coli* (data not shown).

### Improving the cytoplasm redox state and co-expression of a functional electron transport chain promotes violaxanthin production

The activity of ZEP is under tightly regulated in its native hosts. There are six highly conserved cystine residues within ZEP sequences[19]. The oligomeric state of ZEP was shown to be closely related to its activity and tight regulated by redox regulation factors in vivo [19]. This suggested that ZEP has the potential to form intramolecular or intermolecular disulfide bonds, which might be crucial for its activity. The cytoplasm of *Escherichia coli* is highly reduced and, therefore, unfavorable for correct disulfide bond formation. To address this issue, several strategies were used to modify the cytoplasm redox state in the chassis strain. In the *E. coli* cytoplasm, the key disulfide bonds formation-related factors, thioredoxins and glutaredoxins, are maintained in a low-active state by the action of thioredoxin reductase (*trxB*) and glutathione reductase (*gor*), respectively. Cytoplasmic expression of disulfide isomerase (*dsbC*) might also contribute to disulfide bond formation. Thus, a series of chassis strains were obtained by modifying these targets. In the *trxB* knocked-out strain DV22, violaxanthin production was significantly improved to 19.41 ± 3.68 mg/g DCW, about 2-fold that in DV12, with only minimal zeaxanthin and β-carotene formation. The accumulation of antheraxanthin was also eliminated (Fig. 6A, B). However, an additional peak, possibly the by-product 3,6′-Dihydroxy-5,6-epoxy-β,ε-caroten-3′-one, which is produced by the catalytic action of CrtZ, was still detected in the HPLC analysis along with vioxanthin production [20, 21]. The violaxanthin production in the Δ*trxB*Δ*gor* double knocked-out strain DV26 was similar with DV22. However, in other strains, violaxanthin production was not significantly increased (Fig. 6A).

During the epoxidation reaction catalyzed by ZEP, electrons are transferred from FAD to NADPH, and then to ferredoxin. The electron transfer from NADPH to ferredoxin is catalyzed by ferredoxin:NADP^+^ oxidoreductase (FNR) [15]. Furthermore, it was also reported the hydroxylation of β-carotene catalyzed by CrtZ might also need ferredoxin-FNR electron transfer system [22]. It will be important to introduce suitable ferredoxin-FNR system to support both reactions. four plant and four bacteria ferredoxin-FNR systems were evaluated. The ferredoxin-FNR systems were introduced in DV26, and their effects on violaxanthin production were investigated. The results indicated ferredoxin-FNR systems from *Pseudomonas putida*, *Acetivibrio thermocellus*, *Hydrogenobacter thermophilus*, *Spinacia oleracea*, and *Dioscorea alata* did not have a positive effect on violaxanthin production. However, the other three electron transfer systems, namely AciB-AciC from *Acinetobacter sp.*, AtFd-AtFNR from *Arabidopsis thaliana*, and CtFd-CtFNR from *Chlorobaculum tepidum*. could effectively improve violaxanthin production. Among them, the CtFd-CtFNR system (expressed in strain DV37) led to violaxanthin production at 25.28 ± 3.94 mg/g DCW. The content of violaxanthin in the total carotenoids was much increased by strains optimization. This represents the highest violaxanthin production in a metabolically engineered microbial host reported to date.

## Discussion

Carotenoids are among the most widely distributed natural pigments, and more than 1100 carotenoids have been identified so far [23]. Metabolic engineering for the heterologous production of carotenoids in microbial hosts has attracted great interest. However, the oxygenated carotenoids, xanthophylls, which are the most abundant class of carotenoids, are still not widely produced using microbial hosts. In this study, by balancing precursor synthesis in the chassis strain and systematically optimizing the expression of key enzymes, including lycopene β-cyclase (CrtY), β-carotene 3-hydroxylase (crtZ), and zeaxanthin epoxidase (ZEP), we generated a strain producing violaxanthin, an important plant xanthophyll, at 25.28 ± 3.91 mg/g DCW, accounting for 96% of the total carotenoid content in this strain.

The balance of the expression of *crtY*, *crtZ*, and *ZEP* genes is the primary bottleneck of violaxanthin biosynthesis in our strains. Several strategies were tested to optimize the function of these enzymes. There were two major challenges in our optimization research. First, co-expression of *ZEP* with *crtZ* and *crtY* in the heterogenous expression system led to significant reduction of their functions. By fine-tuning the expression of *crtY* and *crtZ* using promoter library, a balanced expression system was obtained. Second, the activity of ZEP involves a series of cofactors, and an efficient electric transfer chain. In line with the fact that ZEP activity is tightly regulated by M-type thioredoxins in *Arabidopsis *[19], we produced a strain with knocked-out *trxB* (encoding thioredoxin reductase), since this enzyme is closely related to the cytoplasm redox potential that affects disulfide bond formation, which plays a crucial role in ZEP stability, and consequently, in violaxanthin production. In addition, ZEP is naturally located in the thylakoid membrane, which makes it difficult to express a fully functional version in *E. coli*. The function of plastid transit peptide-removing ZEP was impacted in our study. The contradictory results compared with other works indicated the heterogenous expression of ZEP is still a challenging problem [10, 15]. Indeed, the carotenoid products might be accumulated in different forms in different microbial hosts. We assumed violaxanthin, similar with other carotenoids, might exist in membrane system of *E. coli* [24]. ZEP might spatially close to membrane-associated violaxanthin during catalyzing epoxidation reaction, therefore its function should be retained in hydrophobic environments. Further research is necessary to clarify the mechanism of ZEP folding and localization in heterologous microbial hosts.

The results of this study demonstrate, for the first time, production of violaxanthin with a high yield in *E. coli*. The violaxanthin yield reported here is the highest obtained in microbial production to date. These results also demonstrate the feasibility of a microbial production route for violaxanthin and other plant xanthophylls.

## Methods

### Chemicals and reagents

Phanta^®^ Max Super-Fidelity DNA Polymerase, the ClonExpress^®^ Ultra One Step Cloning Kit, and other reagents used in molecular biology experiments were purchased from Vazyme Biotech Co., Ltd. (Nanjing, China). The codon-optimized genes were synthesized by GenScript Inc. (Nanjing, China). Standards of carotenoids were purchased from Sigma-Aldrich (Steinheim, Germany). All other chemicals and reagents were purchased from Shanghai ShengGong Bio-chemical Co. Ltd. (Shanghai, China) and were of analytical grade.

### Strains, medium, and DNA manipulations

The *E. coli* strain BW25113 was used as the chassis strain for violaxanthin production, and *E. coli* DH5α was used for plasmid construction. Strains and plasmids used in this study are listed in Table 1. Additional strain and plasmid information is listed in Additional file 1: Table S1.

**Table 1:** Strains and plasmids used in this study

Strain	Genotype	Source
*E. coli* BW25113/F^׳^	*rrnBT*14 Δ*lacZWJ*16 *hsdR*514 Δ*araBADAH*33 Δ*rhaBADLD*78 [F^׳^*proAB lacIqZ*ΔM15 *Tn*10 (*Tetr*)]	CGSC
DL01	BW25113/F’,*lpxM::P*_*araBAD*_*-mvaS-mvaE-mvk-P*_*araBAD*_*-pmk-mvd-idi*, *ldhA::Ptac-crtEBI*	This study
DC01	DL01, pYCA01(P_119_-*PcrtY*(*crtZ* from *Pantoea sp.*), p15A ori)	This study
DZ01	DC01, pMZ01(P_119_-*PcrtZ*(*crtZ* from *Pantoea sp.*), pMBI ori)	This study
DV01	DC01, pMZP01(P_119_-*PcrtZ*,P_119_-*CZEP*(ZEP from *Capsicum annuum*), pMBI ori)	This study
DZ02	DL01, *pflB::P*_*119*_*-PcrtY-PcrtZ*^a^	This study
DV02	DZ02, pMP01(P_119_-*CZEP*, pMBI ori)	This study
DV03	DZ02, pYP01(P_119_-*CZEP*, p15A* ori)^b^	This study
DV04	DZ02, pAP01(P_119_-*CZEP*, ColA ori)	This study
DV05	DZ02, pXP01(P_119_-*CZEP*, p15A ori)	This study
DV06	DZ02, pSP01(P_119_-*CZEP*, pSC101 ori)	This study
DV07	DZ02, pLP01(P_119_-*CZEP*, r6k ori)	This study
DZ12	DL01, pSYZ17(DZ12-crtY-crtZ cluster, pSC101 ori)	This study
DZ13	DL01, *pflB::P*_*DZ1*_*-crtY-P*_*DZ2*_*-crtZ*	This study
DV12	DL01, pSYZ17-CP01 (DZ12-crtY-crtZ cluster, P_119_-*CZEP*, pSC101 ori)	This study
DV13	DZ13, pSP01	This study
DV14	DL01, pSYZ17-CP02 (DZ12-crtY-crtZ cluster, P_119_-*CZEP* (truncating 1–20), pSC101 ori)	This study
DV15	DL01, pSYZ17-CP03 (DZ12-crtY-crtZ cluster, P_119_-*CZEP* (truncating 1–73), pSC101 ori)	This study
DV18	DL01, pSYZ17-AP01 (DZ12-crtY-crtZ cluster, P_119_-*AZEP* (ZEP from *Arabidopsis thaliana*), pSC101 ori)	This study
DV19	DL01, pSYZ17-AP02 (DZ12-crtY-crtZ cluster, P_119_- *AZEP* (truncating 1–57), pSC101 ori)	This study
DV21	DL01, Δ*gor*,pSYZ17-CP01	This study
DV22	DL01, Δ*trxB*,pSYZ17-CP01	This study
DV23	DL01, *pflB::P*_*119*_*-dsbC*,pSYZ17-CP01	This study
DV24	DL01, Δ*gor*,*pflB::P*_*119*_*-dsbC*,pSYZ17-CP01	This study
DV25	DL01, Δ*trxB*,*pflB::P*_*119*_*-dsbC*,pSYZ17-CP01	This study
DV26	DL01, Δ*trxB*,Δ*gor*,pSYZ17-CP01	This study
DV31	DV21,pLFR01 (P_araBAD_-aciB-aciC (from *Acinetobacter sp.*), r6k ori)	This study
DV32	DV21,pLFR02(P_araBAD_-camB-camA (from *Pseudomonas putida*), r6k ori)	This study
DV33	DV21,pLFR03(P_araBAD_-AtFd-AtFnr (from *Arabidopsis thaliana*), r6k ori)	This study
DV34	DV21,pLFR04(P_araBAD_-ActFd-ActFnAB (from *Acetivibrio thermocellus*), r6k ori)	This study
DV35	DV21,pLFR05(P_araBAD_-HtFd-HtFnr (from *Hydrogenobacter thermophilus*), r6k ori)	This study
DV36	DV21,pLFR06(P_araBAD_-SpFd-SpFnr (from *Spinacia oleracea*), r6k ori)	This study
DV37	DV21,pLFR07(P_araBAD_-CtFd-CtFnr (from *Chlorobaculum tepidum*), r6k ori)	This study
DV38	DV21,pLFR08(P_araBAD_-DzSe-DzCpr (from *Dioscorea alata*), r6k ori)	This study
Plasmid^c^		
pLE2SK	*mvaS-mvaE-mvk* cluster	[16]
pSKPMIc	*pmk-mvd-idi* cluster	[16]
pLY122	*crtE-crtB-crtI* cluster	[17]

*CGSC* Coli Genetic Stock Center

^a^P_119_, Ptac promoters used in this study were strong constitutive promoters [18]

^b^The p15A* ori used in the study was derived from a p15A plasmid origin, with a deletion of the 100bp 5′ element within the p15A sequence (GB number: V00309.1), and a higher copy number

^c^Other plasmids used in this study was list in Additional file 1: Table S1

In molecular cloning experiments and seed-culture preparation, *E. coli* strains were grown in Luria-Bertani medium. When necessary, antibiotics were added at the following concentrations: ampicillin, 100 µg/mL; streptomycin, 50 µg/mL; and kanamycin, 50 µg/mL. Auto-induction medium (including 1% tryptone, 0.5% yeast extract, 25 mM Na_2_HPO_4_, 25 mM KH_2_PO_4_, 50 mM NH_4_Cl, 5 mM Na_2_SO_4_, 2 mM MgSO_4_, 0.2× trace metals, 0.5% glycerol, 0.05% glucose, 0.2% lactose, and antibiotics when necessary) [25] was used to induce the expression of pathway genes.

Plasmid construction was performed using standard protocols [26, 27]. Genome editing in *E. coli* was carried out using λ-red-mediated homologous recombination or using P1 transduction from a Keio collection [28–30]. The primers are listed in Additional file 1: Table S2. The source of genes was summarized in Additional file 1: Table S3. The construction was verified by PCR and confirmed by sequencing (Ruibiotech, China).

### Promoter library construction

To construct the promoter library, random sequences were designed in the core motif or between the − 10 and − 35 regions of P_119_ and P_tac_ promoters, which was anticipated to generate a variety of promoters strengths (Additional file 1: Table S4). Degenerate sequences were introduced in the primers when PCR amplifying the promoter fragments. The DNA fragments of two promoters and crtZ were prepared using PCR and assembled with the plasmid pLV01 backbone containing crtY. The obtained plasmid library was then transformed into DL01 and spread on a plate containing ampicillin and L-arabinose to induce the expression of the MVA pathway cluster. Colonies potentially showing high zeaxanthin production (light-yellow colonies) were cultured in 24 deep-well plates in auto-reduction medium. The carotenoid content of each strain was analyzed by HPLC.

### Carotenoids production

Production of carotenoids in flask cultures was carried out using the bioconversion method with optimized conditions. Cells were cultured in auto-induction medium for 16 h, and then collected by centrifugation at 8000 ×*g* for 10 min, washed twice with ice-cold 0.85% NaCl solution, and suspended in 500 µL (OD_600_ = 30) reaction mixture containing 50 mM KH_2_PO_4_/K_2_HPO_4_ buffer (pH 7.0) with 50 mM glucose. The bioconversion was performed at 37 °C with shaking at 200 rpm for 16–20 h. Each bioconversion was repeated at least three times.

### Analysis methods

Cell density was determined by measuring the optical density at 600 nm using an Ultrospec 3000 spectrometer (Pharmacia Biotech, Cambridge, UK). Carotenoid concentrations were determined by HPLC. Cells were collected after bioconversion, washed twice, and then suspended in 1:1 methanol-ethyl acetate at 4 °C for 3–4 h. After centrifugation and filtering through a 0.22 µm membrane, the supernatant was separated by liquid chromatography using a Shimadzu HPLC System (HPLC, LC 20A LabSolutions, Shimadzu Corp., Kyoto, Japan) equipped with a Hypersi 1 BDS C8 3µm column (150×4.6mm). The column temperature was maintained at 30°C and the wavelength of detection was at 450nm. Two mobile phases consisted of A (MeOH) and B (MeOH-0.1M ammonium acetate,7:3, v/v). The elution gradient was as follows(min-%B):0–50; 1–50; 20–30; 25 − 0; 32 − 0; 35–50;45–50, with a flow rate of 1mL/min. The concentration of carotenoids was calculated according to the standard curve. Dry cell weight (DCW) was determined from the optical density at 600 nm (1 OD_600**=**_0.323 g DCW L^− 1^) [31]. Data are means ± SD of three replicates. GraphPad Prism 8 software was used for statistical analysis.

## Supplementary Information

**Additional file 1:** **Table S1.** Additional information of strains and plasmids used in this study. **Table S2.** Primers used for plasmid construction. **Table S3. **Genes used in this study. **Table S4.** Random sequences introduced in P119 and Ptac in promoter library study. **Table S5.** Carotenoids production of different zeaxanthin producing strains. **Table S6.** Carotenoids production of different zeaxanthin producing strains. **Table S7.** Biomass of different strains.

## Data Availability

Not applicable.

## Abbreviations

IPP: Isopentenyl diphosphate
DMAPP: Dimethylallyl diphosphate
MVA: Mevalonate
MEP: Methylerythritol 4-phosphate
FPP: Farnesyl diphosphate
GGPP: Geranylgeranyl diphosphate
RBS: Ribosome binding site
HPLC: High Performance Liquid Chromatography
LB: Luria-Bertani
