# Supplementary material for: Metabolic engineering of Escherichia coli for high-level production of violaxanthin
Source: Microb Cell Fact. 2023 Jun 21;22:115. doi: 10.1186/s12934-023-02098-y (PMC10283192; doi:10.1186/s12934-023-02098-y)
Supplement: Supplementary file 1 — Additional file 1: Table S1. Additional information of strains and plasmids used in this study. Table S2. Primers used for plasmid construction. Table S3. Genes used in this study. Table S4. Random sequences introduced in P119 and Ptac in promoter library study. Table S5. Carotenoids production of different zeaxanthin producing strains. Table S6. Carotenoids production of different zeaxanthin producing strains. Table S7. Biomass of different strains. [file 12934_2023_2098_MOESM1_ESM.docx]

**Table S1 additional information of strains and plasmids used in this study**

| **Strains** | **Description** | **Source** |
| --- | --- | --- |
| DZ03 | DL01,pYCZ01 | This study |
| DZ04 | DL01,pMCZ01 | This study |
| DZ05 | DL01,pLCZ01 | This study |
| DZ06 | DL01,pSCZ01 | This study |
|  |  |  |
|  |  |  |
| **Plasmids** |  |  |
| pYCA01 | P_119_-*crtY*(from *Pantoea sp.*), p15A ori, Amp^r^ | This study |
| pMZP01 | P_119_-*crtZ*(from *Pantoea sp.*),P_119_-*CZEP*, pMBI ori, Kan^r^ | This study |
| pMZ01 | P_119_-*crtZ*, pMBI ori, Kan^r^ | This study |
| pYCZ01 | P_119_-*crtY*,P_119_-*crtZ*, p15A ori, Kan^r^ | This study |
| pMCZ01 | P_119_-*crtY*,P_119_-*crtZ*, pMBI ori, Kan^r^ | This study |
| pLCZ01 | P_119_-*crtY*,P_119_-*crtZ*, r6k ori, Kan^r^ | This study |
| pSCZ01 | P_119_-*crtY*,P_119_-*crtZ*, pSC101 ori, Kan^r^ | This study |
| pMP01 | P_119_-*CZEP*, pMBI ori,Str^r^ | This study |
| pYP01 | P_119_-*CZEP*, p15A ori,Str^r^ | This study |
| pAP01 | P_119_-*CZEP*, ColA ori,Str^r^ | This study |
| pXP01 | P_119_-*CZEP*, p15A variant ori,Str^r^ | This study |
| pSP01 | P_119_-*CZEP*, pSC101 ori,Str^r^ | This study |
| pLP01 | P_119_-*CZEP*, r6k ori,Str^r^ | This study |
| pSYZ17 | *DZ12-crtY-crtZ* cluster, pSC101 ori, Str^r^ | This study |
| pSYZ17-CP01 | *DZ12-crtY-crtZ* cluster, P_119_-*CZEP*, pSC101 ori,Str^r^ | This study |
| pSYZ17-CP02 | *DZ12-crtY-crtZ* cluster, P_119_-*CZEP* (truncating 1-20), pSC101 ori,Str^r^ | This study |
| pSYZ17-CP03 | *DZ12-crtY-crtZ* cluster, P_119_-*CZEP* (truncating 1-73), pSC101 ori,Str^r^ | This study |
| pSYZ17-AP01 | *DZ12-crtY-crtZ* cluster, P_119_-*AZEP*, pSC101 ori,Str^r^ | This study |
| pSYZ17-AP02 | *DZ12-crtY-crtZ* cluster, P_119_- *AZEP* (truncating 1-57), pSC101 ori,Str^r^ | This study |
| pLFR01 | P_araBAD_-*aciB*-*aciC* (from *Acinetobacter sp.*), r6k ori,Kan^r^ | This study |
| pLFR02 | P_araBAD_-*camB*-*camA* (from *Pseudomonas putida*), r6k ori,Kan^r^ | This study |
| pLFR03 | P_araBAD_-*AtFd-AtFnr* (from *Arabidopsis thaliana*), r6k ori,Kan^r^ | This study |
| pLFR04 | P_araBAD_-*ActFd-ActFnAB* (from *Acetivibrio thermocellus*), r6k ori,Kan^r^ | This study |
| pLFR05 | P_araBAD_-*HtFd-HtFnr* (from *Hydrogenobacter thermophilus*), r6k ori,Kan^r^ | This study |
| pLFR06 | P_araBAD_-*SpFd-SpFnr* (from *Spinacia oleracea*), r6k ori,Kan^r^ | This study |
| pLFR07 | P_araBAD_-*CtFd-CtFnr* (from *Chlorobaculum tepidum*), r6k ori,Kan^r^ | This study |
| pLFR08 | P_araBAD_-*DzSe-DzCpr* (from *Dioscorea alata*), r6k ori,Kan^r^ | This study |
| pKD46 | Temperature sensitive vector carrying Red recombinase, Amp^r^ | [22] |
| pCP20 | Temperature sensitive vector carrying FLP recombinase, Amp^r^ | [22] |

**Table S2 primers used for plasmid construction**

| Primers | Sequences (5’3’) | Plasmids |
| --- | --- | --- |
| CRTY-F | ﻿GCTAACAGGAGGAATTAACCATGCCGCGGTATGATCTGATTCTGG | pYCA01 |
| CRTY-R | ﻿TAGTACCAGATCTACCCTCGAGTTATTGCATCGCCTGTTGACGGTGA | pYCA01 |
| CRTZ-F | ﻿GCTAACAGGAGGAATTAACCATGTTGTGGATTTGGAATGCCCTGA | pMZ01 |
| CRTZ-R | ﻿TAGTACCAGATCTACCCTCGAGTTACTTCCCGGGTGGCGCGTCACGC | pMZ01 |
| P119-F | CAGTCGAAAGACTGGGCCTTGGATAACTTGACAGCTAGCTCAGTC | pMZP01  pYCZ01  pMCZ01  pLCZ01  pSCZ01  pSYZ17-CP01  pSYZ17-CP02  pSYZ17-CP03  pSYZ17-AP01  pSYZ17-AP02 |
| TRRN-R | AAATGTTTAAACAAGCTTGAAAGGCCCAGTCTTTCGACTGAGCCT | pMZP01  pYCZ01  pMCZ01  pLCZ01  pSCZ01  pSYZ17-CP01  pSYZ17-CP02  pSYZ17-CP03  pSYZ17-AP01  pSYZ17-AP02 |
| ﻿AZEP-F | ﻿GCTAACAGGAGGAATTAACCATGGGAAGTACACCCTTTTGT | pSYZ17-AP01 |
| AZEP57-F | GCTAACAGGAGGAATTAACCATGAAAGCCGCGACGGCTTTGGTGGAGA | pSYZ17-AP02 |
| ﻿AZEP-R | ﻿TAGTACCAGATCTACCCTCGAGTTATGCGGTTTGCAGCAGTT | pSYZ17-AP01  pSYZ17-AP02 |
| ﻿CZEP-F | ﻿GCTAACAGGAGGAATTAACCATGTATAGCACCGTGTTTTACAC | pMP01  pYP01  pAP01  pXP01  pSP01  pLP01  pSYZ17-CP01 |
| CZEP20-F | ﻿GCTAACAGGAGGAATTAACCATGCAGCTGCCGCTGCTGATCAGCAAGG | pSYZ17-CP02 |
| ﻿CZEP73-F | GCTAACAGGAGGAATTAACCATGCCGCAAAAGAAACTGAAAGTGCTGG | pSYZ17-CP03 |
| ﻿CZEP-R | ﻿TAGTACCAGATCTACCCTCGAGTTACGCGGTACCAACCACCTTG | pMP01  pYP01  pAP01  pXP01  pSP01  pLP01  pSYZ17-CP02  pSYZ17-CP03 |

**Table S3 genes used in this study**

| Gene Name | Origin | Accession No. |
| --- | --- | --- |
| Lycopene β-cyclase | *Pantoea agglomerans* | WP_187491834.1 |
| β-carotene hydroxylase | *Pantoea agglomerans* | ADU76136.1 |
| Zeaxanthin epoxidase | *Capsicum annuum* | XP_047263347.1 |
|  | *Arabidopsis thaliana* | NP_851285.1 |

**Table S4 Random sequences introduced in P119 and Ptac in promoter library study**

| P_119_ promoter | TTGACAGCTAGCTCAGTCCTAGGTATAATG | |
| --- | --- | --- |
| Random sequences introduced in P_119_ promoter | NTNANNGCTAGCTCAGNNCNNGGNANNNTG | |
| Ptac promoter | TTGACAATTAATCATCGGCTCGTATAATG | |
| Random sequences introduced in P_tac_ promoter | NTNANNATTAATNNNNGGCTCGNANNNTG | |
|  |  | Parent promoter |
| Promoter of *crtY* in P12D3 (DZ12) | CTTACGGCTAGCTCAGATCTCGGTATTGTG | P_119_ |
| Promoter of *crtZ* in P12D3 (DZ12) | TTGACAGCTAGCTCAGCGCTTGGTACAATG | P_119_ |
| Promoter of *crtY* in P1B5 | TTGATGGCTAGCTCAGTGCTAGGGATTATG | P_119_ |
| Promoter of *crtZ* in P1B5 | TTGACATGCAATCTGCGGCTCGTATAGTG | P_tac_ |

-10 and -35 regions of the promoters were underlined.

**Table S5 carotenoids production of different zeaxanthin producing strains**

| **Strains** | **Carotenoids production mg/g DCW** | | |
| --- | --- | --- | --- |
|  | **lycopene** | **β-carotene** | **zeaxanthin** |
| DZ01 | 8.90±1.33 | 32.36±1.73 | 7.08±1.50 |
| DZ02 | 24.40±1.15 | 12.39±1.40 | 17.08±1.71 |
| DZ03 | 18.26±1.05 | 20.03±1.62 | 5.35±0.70 |
| DZ04 | 9.34±1.20 | 27.27±1.77 | 11.66±0.95 |
| DZ05 | 17.71±1.71 | 10.11±2.03 | 13.91±1.05 |
| DZ06 | 13.08±1.31 | 19.50±1.01 | 15.21±1.34 |

**Table S6 carotenoids production of different zeaxanthin producing strains**

| **Strains** | **Carotenoids production mg/g DCW** | | | | |
| --- | --- | --- | --- | --- | --- |
|  | **lycopene** | **β-carotene** | **zeaxanthin** | **violaxanthin** | **antheraxanthin*** |
| DV12 | 0.78±0.17 | 1.61±0.34 | 4.25±0.65 | 11.49±0.12 | 5.77±0.20 |
| DV21 | 0.45±0.38 | 1.33±0.15 | 3.36±0.32 | 12.79±1.25 | 5.60±0.35 |
| DV22 | 0 | 0.72±0.41 | 0.36±0.06 | 19.41±3.68 | 0.99±0.16 |
| DV23 | 0.86±0.25 | 2.66±0.07 | 2.54±2.03 | 9.86±2.66 | 4.63±1.26 |
| DV24 | 0.91±0.42 | 1.66±0.19 | 3.90±0.71 | 11.67±0.34 | 2.19±0.02 |
| DV25 | 0.49±0.13 | 2.06±0.80 | 0.97±0.09 | 11.27±2.90 | 0 |
| DV26 | 0 | 0.73±0.30 | 0.57±0.52 | 19.41±1.01 | 0.96±0.06 |

*Calculated using violaxanthin standard curve.

**Table S7 Biomass of different strains**

| **Strains** | O.D._600_ after induction | O.D._600_ after bioconversion | Results of | **Strains** | O.D._600_ after induction | O.D._600_ after bioconversion | Results of |
| --- | --- | --- | --- | --- | --- | --- | --- |
| DL01 | 8.16±0.66 | 27.16±1.30 | Fig.2 | DV18 | 7.33±0.30 | 28.59±3.54 | Fig.5 |
| DC01 | 7.93±0.79 | 26.93±2.55 | Fig.2 | DV19 | 8.50±0.75 | 26.39±4.53 | Fig.5 |
| DZ01 | 7.05±0.42 | 29.05±4.88 | Fig.2 | DV21 | 9.17±0.53 | 25.63±1.72 | Fig.6 |
| DZ02 | 7.34±0.59 | 27.34±2.50 | Fig.3 | DV22 | 8.67±0.67 | 28.11±2.65 | Fig.6 |
| DZ12 | 7.52±0.88 | 27.53±3.60 | Fig.5 | DV23 | 7.04±0.82 | 29.72±4.71 | Fig.6 |
| DZ13 | 8.21±0.84 | 28.71±1.90 | Fig.5 | DV24 | 7.85±0.91 | 30.21±3.90 | Fig.6 |
| DV01 | 8.41±0.61 | 25.81±2.54 | Fig.2 | DV25 | 7.96±0.48 | 29.45±4.88 | Fig.6 |
| DV02 | 8.01±0.75 | 29.88±3.06 | Fig.3 | DV26 | 8.01±1.04 | 26.03±2.66 | Fig.6 |
| DV03 | 7.18±0.43 | 30.18±0.85 | Fig.3 | DV31 | 7.18±0.55 | 27.18±4.52 | Fig.6 |
| DV04 | 7.72±0.87 | 28.02±4.37 | Fig.3 | DV32 | 6.83±0.52 | 31.02±2.30 | Fig.6 |
| DV05 | 6.99±0.90 | 26.98±1.90 | Fig.3 | DV33 | 8.45±0.66 | 26.80±1.15 | Fig.6 |
| DV06 | 7.57±1.20 | 25.57±2.06 | Fig.3 | DV34 | 7.82±0.42 | 25.35±3.20 | Fig.6 |
| DV07 | 8.34±0.66 | 27.06±3.25 | Fig.3 | DV35 | 6.86±0.97 | 26.34±3.59 | Fig.6 |
| DV12 | 8.56±0.67 | 27.82±2.33 | Fig.5 | DV36 | 8.09±0.73 | 29.07±2.09 | Fig.6 |
| DV13 | 7.63±0.98 | 28.03±2.10 | Fig.5 | DV37 | 7.90±0.36 | 27.63±1.70 | Fig.6 |
| DV14 | 7.93±0.25 | 26.93±1.99 | Fig.5 | DV38 | 7.54±0.45 | 30.55±4.62 | Fig.6 |
| DV15 | 7.30±0.63 | 25.55±3.33 | Fig.5 |  |  |  |  |
